# Attention-Based Graph Neural Network for Label Propagation in Single-Cell Omics

**DOI:** 10.3390/genes14020506

**Published:** 2023-02-16

**Authors:** Rahul Bhadani, Zhuo Chen, Lingling An

**Affiliations:** 1 Department of Electrical & Computer Engineering, The University of Arizona, Tucson, AZ 85721, USA; 2 Interdisciplinary Program in Statistics and Data Science, The University of Arizona, Tucson, AZ 85721, USA; 3 Department of Biosystems Engineering, The University of Arizona, Tucson, AZ 85721, USA; 4 Department of Epidemiology and Biostatistics, The University of Arizona, Tucson, AZ 85721, USA

**Keywords:** single-cell, transcriptomics, scRNA-seq, graph neural network, classification, label propagation, neural network

## Abstract

Single-cell data analysis has been at forefront of development in biology and medicine since sequencing data have been made available. An important challenge in single-cell data analysis is the identification of cell types. Several methods have been proposed for cell-type identification. However, these methods do not capture the higher-order topological relationship between different samples. In this work, we propose an attention-based graph neural network that captures the higher-order topological relationship between different samples and performs transductive learning for predicting cell types. The evaluation of our method on both simulation and publicly available datasets demonstrates the superiority of our method, scAGN, in terms of prediction accuracy. In addition, our method works best for highly sparse datasets in terms of F1 score, precision score, recall score, and Matthew’s correlation coefficients as well. Further, our method’s runtime complexity is consistently faster compared to other methods.

## 1. Introduction

Single-cell RNA sequencing (scRNA-seq) technology has proven to advance biological research in an unprecedented way by sequencing cells at a single-cell resolution [1]. Compared to the bulk RNA sequencing, scRNA-seq allows for studying biological cell samples with much greater detail. An important problem in scRNAseq research is the identification of cell types that can assist researchers to conduct further analyses such as distinguishing diseased cells from healthy cells. There are two main ways to annotate cells: (i) unsupervised learning, that is using cell clustering techniques and then finding marker genes specific to a cluster and annotate cells belonging to that cluster as per the ontological functions of their genes [2]; and (ii) supervised or semi-supervised learning techniques where we are given cell-samples with true cell-types (or labels), perform model-fitting, machine-learning or deep-learning techniques and perform label-transfer on unseen cell-samples [3]. Clustering techniques are unsupervised—they annotate cell types based on the underlying structure of the dataset and no knowledge of ground truth cell types is required in creating a model. However, unsupervised learnings suffer from two major drawbacks: (i) difficulty of interpretability, as they are more generic in nature; and (ii) lack of clear evaluation metrics in the absence of ground truth data. In such cases, we require the supervision of domain experts who need to interpret the result by close inspection either through their own experience or by means of additional sources. Supervised or semi-supervised learning techniques require the presence of ground truth cell-types for all or a portion of the dataset to create a model that can be used for making predictions on an unseen dataset. However, it is not always possible to obtain ground truth data. Further, such methods may not be robust to outliers and noise. Examples of clustering techniques available in the literature are Seurat [4], SC3 [5], ClonoCluster [6], and IsoCell [7]. Among supervised learning methods, some notable methods include SingleR [8], Seurat V3 [9], Chetah [10], and scMAP [11].

Seurat [4] uses community detection algorithms such as Louvain [12] to create clusters. The SC3 method uses a cell similarity matrix and k-means for clustering [5]. Some novel methods such as ClonoCluster use clonal information along with transcriptomics to calculate hybrid clusters [6]. The IsoCell method uses alternative splicing by integrating isoform-level expression and gene-level expression to perform clustering on single-cell data [7]. Reference-based methods may fall into the category of supervised or semi-supervised learning methods where a model is constructed based on a reference dataset with true cell types or known labels. Using the constructed model, predictions can be made on an unseen dataset. Seurat V3 [4] constructs a canonical correlation matrix [13] and finds anchor points to create a network model that can be used for making predictions on an unseen dataset. scMAP provides a method where transcriptomics data corresponding to individual cells are projected onto the cell types of transcriptomics data obtained from another experiment. scMAP includes two sub-methods: scMAP-clusters and scMAP-cell. Given a new cell, the task is to project the new cell onto a reference dataset and look for either a cluster most similar to the new cell (scMAP-cluster), or an individual cell similar to the new cell (scMAP-cell) [11]. Another reference-based method called Chetah uses a reference dataset with known cell types to create a classification tree, and the correlation is performed iteratively between a new dataset and classification tree to assign cell labels. SingleR uses reference transcriptomic datasets with given cell types to infer the cell types of a query dataset based on similarity measures.

However, the methods discussed above only consider the first-order direct relationship between cells and ignore the higher-order topological relationship. Higher-order topological relationships, such as non-linear interactions between features, may be important for correctly classifying or clustering the samples. All of the methods discussed above do not take into account non-linear relationships in the dataset. Further, ClonoCluster requires additional information in the presence of a clonal subpopulation, which might not be available all the time. The IsoCell method requires isoform-level expression and fails to generalize. scMAP struggles in a situation where datasets exhibit high noise. In the case of SingleR, the choice of similarity measures affects the prediction on an unseen dataset.

Higher-order topological relationships can be captured in a model using a graph neural network. Traditionally, Artificial Neural Networks (ANN) have employed linear relationships in the given dataset of interest to find patterns, perform model-fitting, make predictions, and perform statistical inferences. However, ANN works with datasets such as matrices, vectors, and linear data structures and is not suited for datasets with a hierarchical structure such as trees, heaps, graphs, hypergraphs, hash tables, etc. For a hierarchical data structure such as a graph, graph neural networks (GNN) are well suited to perform learning [14,15]. GNNs have been shown to be effective at tasks such as node classification, link prediction, and graph classification, and have been applied to a wide range of domains including computer vision, natural language processing, electrical engineering, and bioinformatics [16,17,18,19].

GNN is based on the idea that the characteristics of a node are determined by its neighboring nodes and the connections between them. To illustrate this, consider the fact that if a node were to lose all of its neighboring connections, it would also lose its meaning and context. In other words, a node’s neighbors and connections to them are crucial in defining its characteristics [20]. Since graphs are unstructured and any representation of a graph can lead to very high dimensional matrices, it is important to calculate low-dimensional representations. Such representations are called embedding. Since usually a graph contains thousands of nodes, we are interested in calculating low-dimensional vector representations of nodes, called node embedding. A few popular methods to calculate node embedding are message passing [21], random projection [22], and Node2Vec [23].

There are several ways to implement a graph neural network such as Graph Convolutional Networks (GCN) [24], Graph Autoencoders (GAE) [25], and Attention-based Graph Neural Networks (AGN) [26], to name a few. GCNs use a variation of the convolution operation, known as graph convolution, to operate on the graph structure. They are typically used for node classification and graph classification tasks. GAEs are a type of unsupervised GNN that are trained to reconstruct the graph structure by encoding and decoding the graph. AGN uses attention mechanisms to weigh the importance of the neighboring nodes for each node. The attention mechanism provides a way to learn a dynamic and adaptive local representation of the neighborhood to achieve better predictions. AGNs are typically used for node classification, graph classification, and other graph-related tasks.

In this work, we propose scAGN, a method that includes an attention-based graph neural network for cell-type detection on a scRNA-seq dataset by means of label-propagation. The method uses transductive learning for label transfer to query datasets given a reference dataset. Transductive learning is a learning method in which both the training and testing datasets are used during the learning phase. The model looks for patterns in the combined dataset of training and testing data and then uses this information to make predictions for the unlabeled testing data points. We perform transductive training on a number of publicly available single-cell transcriptomics datasets using the scAGN method. Our analyses show that the scAGN method allows for accurate prediction of cell-labels by knowledge transfer from a reference dataset to a query dataset. As a baseline, we compare the scAGN method with the previously proposed methods Chetah, Seurat V3, scmap-cell, scmap-cluster, and singleR based on a number of metrics such as prediction accuracy, precision score, recall score, F1 score, and Matthew’s correlation coefficient. We demonstrate that, overall, scAGN outperforms the baseline methods. We provide the source code of our method and an online repository of the dataset used.

## 2. Materials and Method

To evaluate the effectiveness of our proposed method in cell-type identification, we used both simulated datasets and real datasets that have been published and are freely accessible. In this section, we first describe datasets used for assessing the performance of scAGN and the performance comparison with existing methods. Next, we explain the scAGN method in detail including the data preparation and graph construction.

### 2.1. Simulated Datasets

A scRNA-seq simulator, Splatter [27], is used to generate simulated data. To mimic real scRNA-seq count data, which are usually very sparse (i.e., with excessive zeros), we used high dropout rates in the simulation. In scRNA-seq, dropout is used to denote the phenomenon where certain genes are not detected or are under-represented in certain cells due to limitations in the sequencing technology. This may lead to missing data in the scRNAseq dataset, which can introduce noise and bias into the downstream analysis [28,29,30]. These missing data are replaced with zero in the standard protocols for scRNA-seq data analysis. Each such dataset is represented as a cell–gene matrix, with genes as columns and cell samples as rows. In our simulation study, each simulated dataset (called count matrices) contains 1000 cells and 800 genes with equiprobable four cell types. The sparsity of each dataset was roughly 95%. Here, sparsity is defined as the percentage of zeros in the count matrix. We generated 50 replicates of simulated datasets under this setting (i.e., 95% zeros and equiprobable four cell types)). Further, to investigate the impact of imbalanced cell classes, we generated 50 replicates of simulated datasets with 95% sparsity and four imbalanced cell classes (i.e., types): 5%, 15%, 35%, and 45%. Each class represents a cell type. More details about the generation of the simulated dataset can be found in the supplementary material.

**Figure 1:**
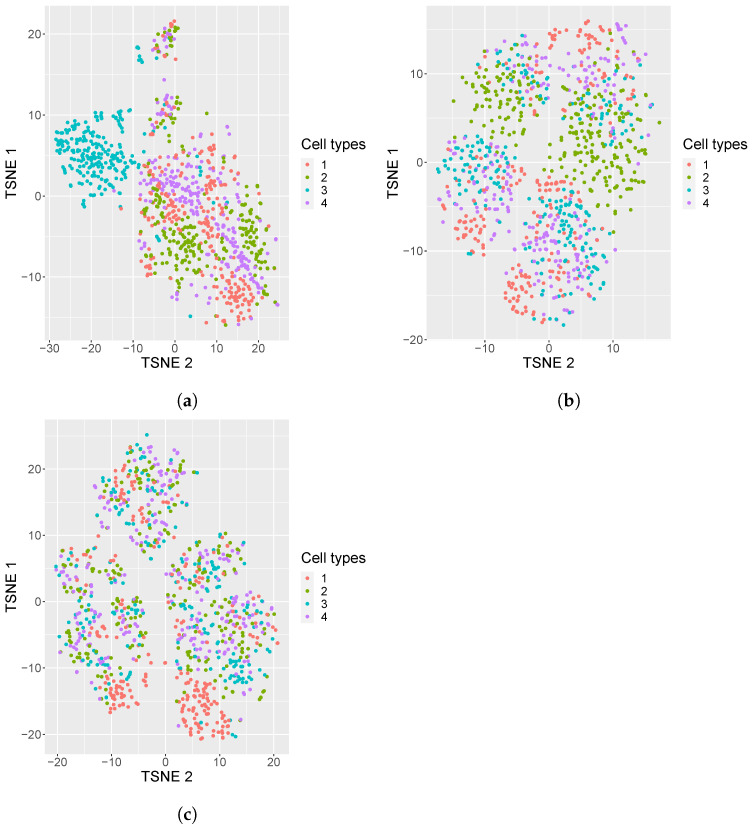
tSNE plots of simulated datasets with varying sparsity or composition of cell types. (**a**) tSNE plots of simulated datasets with 80% sparsity, four equiprobable cell types; (**b**) 95% sparsity, four equiprobable cell types; (**c**) 95% sparsity, unequal number of cell samples from each cell type (i.e., 5%, 15%, 35%, 45%).

In addition, we also generated 50 replicates of simulated datasets with a medium sparsity of 80% containing 4 equiprobable classes. A visualization of these simulated datasets under various settings is shown in Figure 1: one dataset with 80% sparsity having equiprobable classes, and two datasets, one with the sparsity of 95% with equiprobable classes and the second with imbalanced classes. From the visualization, it is evident that the cell types in the noisy data are mixed, which brings in a challenge for cell type identification.

### 2.2. Real Datasets

For scAGN’s performance evaluation, we used 12 real datasets, summarized in Table 1. The datasets were obtained from publicly available repositories. The characteristics of each dataset and the technology used to sequence each dataset are provided separately in the next section. Overall, the datasets represent a mix of sequencing technology consisting of Smart-seq2, 10x, Seq-well, etc. In addition to healthy cells, we also studied cell-type detection for cancerous cells as well as from COVID-19 patients obtained from the COVID-19 atlas [31].

We first consider transcriptomics data representing healthy cells: (i) TM, (ii) Tasic, (iii) PBMC 68K, and (iv) Mouse Retina. The TM dataset was obtained from GSE109774, consisting of transcriptomics of 20 organs and tissues from Mus musculus [32]. A total of 54,865 cells and 2000 genes consisting of 55 cell types were used in our study. The Tasic dataset contains transcriptomics from adult mouse cortical cells consisting of 1679 cells and 2000 genes [33] with 17 cell types. PBMC 68K is a slightly larger dataset consisting of 68,579 cells and 2000 genes with 10 cell types of human peripheral blood mononuclear cells obtained from SRP073767. The Mouse Retina dataset consists of 27,499 cells and 2000 genes with 19 cell types obtained from GSE81904 [34] (All four datasets together can be obtained from https://www.synapse.org/#!Synapse:syn26524750/files/ (accessed on 25 December 2022)).

Next, we consider cancer cells. It is known that T cells play a crucial role in the immunotherapy of cancer. Single-cell transcriptomics data obtained from sequencing T cells of colorectal cancer can help in identifying healthy cells from cancerous cells. We used the dataset GSE108989 from [35] that was sequenced by the Smart-seq2 protocol. The GSE108989 dataset contains 11,138 cells obtained from 12 colorectal patients with 23,459 genes. The ground truth data contain 5 broad cell types that were used for training our graph neural network. The second cancer dataset GSE118389 used in our study was obtained from [36]. GSE118389 contains a transcriptomics dataset from triple-negative breast cancer cells with 1534 cells and 21,785 genes. In this case, the cell types are six different disease states. Cells from six triple breast cancer tumors were obtained using the Smart-seq2 protocol. The third cancer dataset used, GSE72056, regards melanoma cancer that was obtained by sequencing melanoma tumors using the Smart-seq2 protocol by Tirosh et al. [37]. GSE72056 contains 4513 cells and 23,690 genes. The authors of the dataset annotated 7 different cell types, which we used as ground truth labels in our study. The next cancer dataset in our study is GSE98638, which contains transcriptomics data obtained by sequencing liver cancer cells using Smart-seq2 protocol [38]. GSE98638 dataset contains 5063 cells and 23,459 genes with 12 different cell types. The final dataset in this series is lung cancer dataset GSE99254, which was obtained by sequencing samples obtained from non-small cell lung cancer patients using a Smart-seq2 protocol [39]. The GSE99254 dataset contains 12,346 cells and 23,459 genes with 19 cell types.

Further, we also consider three COVID-19 datasets. In early 2020, an outbreak of a novel coronavirus (SARS-CoV-2) happened in Wuhan of Hubei Province, China. The virus quickly spread throughout the globe through air travel, cruise ships, and other modes of transportation. Due to the extreme nature of the outbreak, many scientists have studied transcriptomics data from COVID-19 patients extensively. In this study, we used three COVID-19 datasets. The first one, which we call blish_pbmc, contains 44,721 cells and 26,361 genes with 16 cell types. The dataset was sequenced using the seq-well protocol [31]. The second dataset, called nasal_epithelia, was sequenced from nasopharyngeal samples. The samples were obtained from 35 COVID-19 patients. Seq-Well was used for sequencing the samples to obtain transcriptomics consisting of 32,588 cells and 32,871 genes with 17 different cell-types [40]. The third dataset used in our study is villani_mgh, which consists of 59,506 cells and 24,179 genes with 16 cell types (All three datasets were obtained from the COVID19 atlas project https://www.covid19cellatlas.org/ (accessed on 25 December 2022)). A summary of these datasets including sparsity is provided in Table 1.

### 2.3. Data Preparation

Each input dataset is represented as a cell–gene matrix with genes as columns and cell samples as rows, assuming *g* genes and *c* cells. Genes are considered features in transcriptomics datasets as they characterize a cell sample. Such a dataset is also referred to as a feature dataset. Consider the feature dataset $F\in R^{g\times c}$ as the input to the scAGN pipeline. We construct the reference and query dataset by splitting the cells in F into the p:1−p ratio. If there are L number of known labels (i.e., cell types or classes) for the feature dataset, we construct a reference and query dataset so that each of the L labels is also split into the ratio p:1−p for balanced training representing the ratio of each label in reference and query datasets. Generally, the size of the reference dataset is larger than the one of the query set, so the *p* needs to be more than 0.5. We recommend 0.75 for *p*, and the ratio between the sizes of the two datasets is 3:1.

### 2.4. Graph Construction

Consider the reference feature dataset to be $F_{R}\in R^{g\times r}$ and the query dataset to be $F_{Q}\in R^{g\times q}$, where *g* is the number of features (or genes) *r* is the number of cells in the reference dataset, and *q* is the number of cells in the query dataset. Usually, there is a large number of genes in a typical single-cell RNA study and only a portion of the genes show differences between cell types. Thus, we need to identify the most variable genes in the reference dataset. The Analysis of Variance (ANOVA) technique was applied to each gene across its expression values in all cells [41,42], then we further applied a Bonferroni correction for selecting the top genes (assume *m* genes out of *g* genes) [43]. We use the selected *m* genes for both reference and query datasets. Thus, we end up with the reference dataset as $F_{R}\in R^{m\times r}$ and the query dataset as $F_{Q}\in R^{m\times q}$. For this work, we select m=2000 genes or all of the genes if the total number of genes is less than 2000 genes in the dataset.

For graph construction, we use canonical correlation analysis (CCA) [13], which reveals a shared gene-correlation structure between the reference and query datasets (Canonical Correlation Analysis (CCA) is a statistical technique used to analyze the relationships between two sets of variables. The goal of CCA is to find linear combinations of the variables in each set (called canonical variables) that have a maximum correlation with each other. The correlation between the two sets of canonical variables is called the canonical correlation coefficient. CCA is often used in the field of multivariate statistics to identify patterns or relationships between two or more sets of variables. It can be used for various applications, such as dimensionality reduction, feature selection, and pattern recognition [13]). A shared gene-correlation structure is used to project reference and query datasets into the same low-dimensional space. In these cases, the main goal of CCA is to find a linear combination of gene features across the reference and query datasets that are maximally correlated. The CCA algorithm returns a linear combination of genes as a basis vector that can be understood as meta-genes. This requires solving the following objective:

(1) $$\begin{array}{c} \max{\;}_{u,v}\;u^{⊤}{\left(F_{R}\right)}^{⊤}F_{Q}{v\;\mathrm{such}\;\mathrm{that}\;||u||}_{2}^{2}\leq {1,||v||}_{2}^{2}\leq 1, \end{array}$$

The graph construction is performed in the following steps:

- First, we transform the dataset by subtracting with the gene-wise mean and dividing by the gene-wise variance.
  (2) $$\begin{array}{c} \tilde{f_{ij}}=\left(f_{ij}-\mu_{i}\right)/\sigma_{i} \end{array}$$
  where $f_{i}$ is an entry in the F matrix, $\mu_{i}$ is the mean of gene expression of gene *i* for all cell samples and $\sigma_{i}$ is the standard deviation for gene *i* for all cell samples.
- We calculate $K={\left(F_{R}\right)}^{⊤}F_{Q}$, which can be decomposed using singular value decomposition (SVD) as $K=\Gamma \Delta \Lambda^{⊤}$ where $\Gamma =[\gamma_{1},\gamma_{2},\cdots ,\gamma_{k}]$, $\Lambda =[\lambda_{1},\lambda_{2},\cdots ,\lambda_{k}]$, and $\Delta =[\delta_{1}^{1/2},\delta_{2}^{1/2},\cdots ,\delta_{k}^{1/2}]$
- We can obtain canonical correlation vectors u and v as left and right singular vectors for up to *k* components, where *k* is a user choice. In this work, we select k=5.
- Alignment of two canonical vector u and v is performed using dtw R package [44].
- Based on the cell’s embedding on aligned basis vector u and v, a minimum nearest neighbor graph is constructed where adjacency matrix A has an entry 1 if cell *i* is one of the nearest neighbors of cell *j* and vice-versa, otherwise the entry is 0. Adjacency matrix A is first converted to a sparse edge indices format and then reverse edges are added to make the graph undirected for input to the Attention-based Graph Neural Network. It is this sparse edge indices format that we refer to as a graph in our method.
- We construct two kinds of graphs: one using $F_{R}$ and $F_{Q}$, and another one using two sets of $F_{Q}$. These two graphs after converting to the sparse edge-index format are appended together to obtain F and indices are updated based on the indices of the concatenated feature matrix, $F_{R}$ and $F_{Q}$.

### 2.5. Feature Processing

Here, we provide details on constructing training, test, validation, and prediction sets from feature dataset F. As a reminder, F is a cell × gene matrix with genes as features. We first calculate a diagonal matrix:

(3) $$\begin{array}{c} G=\left[\begin{array}{c} {\left(\sum_{1}F\right)}^{-1},\;\;0\;\;,\cdots ,\;\;0\;\;\\ \;\;0\;\;,{\left(\sum_{2}F\right)}^{-1}\cdots ,\;\;0\;\;\\ \;\;0\;\;,\ddots ,\cdots ,\;\;0\;\;\\ \;\;0\;\;,\cdots ,\;\;0\;\;,{\left(\sum_{r+q}F\right)}^{-1} \end{array}\right] \end{array}$$

where $\sum_{r+q}$ is the sum of genes *i* for all cell samples. Using G, we feature-normalize the matrix F as $\tilde{F}=G\cdot F$. $\tilde{F}$ is split into a training, test, validation, and prediction set (the prediction set is also called an independent set in the literature). The length of the training set is the same as the number of cell samples in $F_{R}$, while the prediction set is sampled from F consisting of nodes corresponding to the query dataset. Overall, 80% of the reference data are used as training while 10% each are used as a test and validation set.

### 2.6. Message-Passing Technique for Graph Neural Network

**Figure 2:**
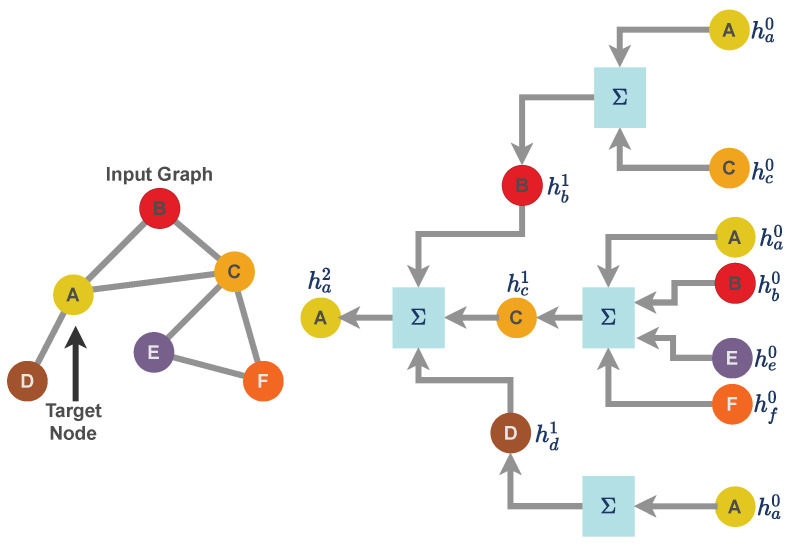
Neighborhood aggregation for message-passing techniques for a Graph Neural Network. A graph neural network can be represented as a layer that consists of two mathematical operators: (i) aggregation function and (ii) update function. For the graph illustrated in this figure, we aggregate the features of node A up to the depth of two layers. Similarly, aggregation can be performed for each node by averaging features over its out-degree and then receiving others’ messages by averaging over their in-degrees, which is why the overall scheme is called the message-passing technique.

For the work presented in this paper, we use a specific implementation of a graph neural network called a message passing neural network [21]. Unlike a grid structure or linear data structure, graph data structures have arbitrary topology and there is no fixed node ordering or reference point. As a result, graph-like data structure uses a neural message passing technique for exchanging features between nodes and to update node embedding from layer to layer. Consider a graph M≡f(F,E) as a graph neural network model where *f* is a generic neural network function with F as the feature matrix and E as the sparse edge representation of a graph. Further, consider $h_{i}^{\left(t\right)}$ to be a node embedding for the node i∈F with F representing the feature dataset in the form of vertices. N(j) is the set of neighbors of node *j*. Message passing is carried out through the neighborhood aggregation of node features as illustrated in Figure 2. The message passing [45,46] scheme can formulated as

(4) $$\begin{array}{cc} h_{i}^{\left(\ell +1\right)} & ={\mathrm{UPDATE}}^{\left(\ell \right)}\left(h_{i}^{\left(\ell \right)},{\mathrm{AGGREGATE}}^{\left(k\right)}\left(h_{j}^{\left(\ell \right)},\forall j\in N\left(i\right)\right)\right)\\ & ={\mathrm{UPDATE}}^{\left(\ell \right)}\left(h_{i}^{\left(\ell \right)},m_{N(i)}^{\left(\ell \right)}\right) \end{array}$$

where AGGREGATE function takes as input the set of embeddings of the nodes in *j*’s graph neighborhood N(j) and generates a message m. UPDATE combines the message with the previous embedding of node *j* to generate the updated embedding. UPDATE and AGGREGATE are mathematically differential functions (or neural networks). The AGGREGATE function aggregates information from its local neighborhood. m is the message that becomes aggregated using the neighbor nodes’ feature of node *i*. The superscript of h is used to denote iterations of message passing, which are effectively the layers of the graph neural network.

### 2.7. Architecture of Attention-Based Graph Neural Network

The purpose of the graph neural network model M is to estimate the probability of node *j* of a graph with edge representation E belonging to one of the L labels. Assume that in the graph with edge representation E, the neighborhood of node *j* is denoted by N(j). In an attention-based graph neural network, the first layer is defined by

(5) $$\begin{array}{c} h^{\left(1\right)}=\mathrm{ReLU}\left(FW^{0}\right) \end{array}$$

where $W^{0}$ is the initial weight. The consecutive layer is defined by

(6) $$\begin{array}{c} h^{\left(t+1\right)}=P^{\left(t\right)}h^{\left(t\right)} \end{array}$$

where $P^{\left(t\right)}$ is the propagation matrix. Each row of $h^{\left(t+1\right)}$ can be written as

(7) $$\begin{array}{c} h_{i}^{\left(t+1\right)}=\sum_{j\in N(i)\cup \{i\}}P_{ij}^{\left(t\right)}h_{j}^{\left(t\right)} \end{array}$$

where $P_{i}^{\left(t\right)}=\mathrm{softmax}\left({\left[\beta^{t}\cos\left(h_{i}^{\left(j\right)},h_{j}^{\left(j\right)}\right)\right]}_{j\in N(i)\cup \{j\}}\right)$ and $\cos\left(a,b\right)=a^{⊤}b/\left(||a|\right|\cdot \left||b||\right)$, and ||·|| is $L_{2}$ norm. Each layer is parameterized by the $\beta_{t}$ value. The final layer has weight $W^{1}$, using which we can calculate a prediction using a softmax function as follows:

(8) $$\begin{array}{c} M=\mathrm{softmax}(h^{\left(\ell +1\right)},W^{1}) \end{array}$$

where the number of layers is *ℓ*. In this network architecture, we define **attention** as

(9) $$\begin{array}{c} P_{ij}^{\left(t\right)}=\frac{1}{\sum_{j\in N(i)\cup \{j\}}e^{\beta^{\left(t\right)}\cos\left(h_{i}^{\left(j\right)},h_{j}^{\left(j\right)}\right)}}\cdot e^{\beta^{\left(t\right)}\cos\left(h_{i}^{\left(j\right)},h_{j}^{\left(j\right)}\right)} \end{array}$$

which denotes how similar the two cell samples *i* and *j* are. The attention selects neighbors with the same class to be more relevant.

### 2.8. scAGN Method

**Figure 3:**
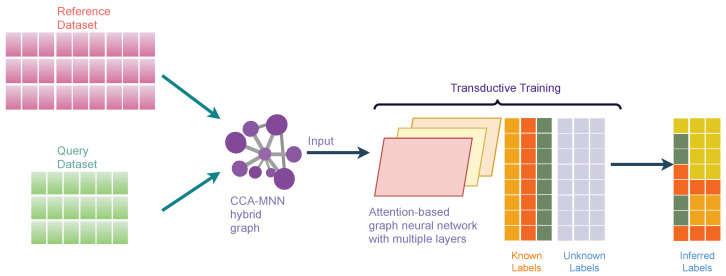
An illustration of scAGN. It shows the concept behind transductive training on the CCA-MNN graph using Attention-based Graph Neural Network architecture. The reference and query datasets are jointly processed to create a feature set that is used to calculate CCA-MNN hybrid graph representation in a sparse edge format. The graph is used as an input along with true labels for reference datasets to the attention-based graph neural network for training. The trained model can be used for making a prediction on an unseen dataset.

Attention-based Graph Neural Network (AGN) is a type of graph neural network that removes the intermediate fully-connected layers and replaces the propagation layers with attention mechanisms that respect the structure of the graph. This attention mechanism allows us to learn an adaptive and dynamic local summary of the neighborhood to achieve more accurate predictions. Our proposed method, scAGN, employs AGN architecture where single-cell omics data are fed after batch-correction using canonical correlation analysis and mutual nearest neighborhood (CCA-MNN) [47,48] as explained above. scAGN uses transductive learning to infer cell labels for query datasets based on reference datasets whose labels are known or have been annotated by experts. The illustration of scAGN is shown in Figure 3. We split the dataset in the ratio of 3:1 (p=0.75), where 75% of the dataset acts as a reference dataset and 25% of the dataset acts as a query dataset. We perform transductive learning on a graph constructed using CCA-MNN, where some labels (such as cell types, diseased/unhealthy cells, etc.) are known and some are not known (Figure 3).

### 2.9. Implementation and Model Training

We use Pytorch-Geometric [49] for the implementation of AGNN architecture. Our implementation uses a user-specified multiple attention layer. The first layer is a linear layer that applies affine transformation. The subsequent layers are the attention layer with the very first attention layer having β=1. β values for subsequent attention layers are randomly initialized between 0 and 1. The last layer is specified as a linear layer followed by a softmax function for label prediction. The last layer is an output layer with size *n* where *n* is the number of classes or cell types. The output layer predicts the probability of cell types of each cell as $p=[p_{1},p_{2},\cdots ,p_{n}]$. The predicted cell type for each cell is the class *i* for which we obtain the highest probability, i.e., ${\hat{y}}_{i}=\mathrm{argmax}\left(p\right)$. For a given true class $y_{i}$, we compute the loss as a negative log-likelihood as follows:

(10) $$\begin{array}{c} L=-\log(p_{y_{i}}) \end{array}$$

where $p_{y_{i}}$ is the output probability of cell-type $y_{i}$.

We train AGNN architecture with negative log-likelihood as a loss function with Adam optimizer. Further, we use a step learning rate where the learning rate is decreased with epochs of training. Step learning is parametrized by two parameters: step size and γ. We use a step size of 10 and γ=0.8, which means the learning rate is reduced by 80% every 10 steps. We also utilize an early-stopping mechanism for terminating the training procedure if no improvement in validation accuracy is achieved. Early stopping size is parametrized by patience value *v*, for which we use v=2000, which means if no improvement is made for the next *v* epochs, training stops.

### 2.10. Performance Evaluation

We compare the proposed method with five baseline methods: (i) Seurat V3, (ii) SingleR, (iii), scMAP-cluster, (iv) scMAP-cell, and (v) Chetah. We use five evaluation metrics for comparing the performance of our method against baseline methods: (i) prediction accuracy, (ii) precision score, (iii) recall score, (iv) F1-score [50], and (v) Matthew’s correlation coefficient [51]. Consider the total number of labels to be *N*. Further, consider TP as true positive, FP as false positive, TN as true negative and FN as false negative. Thus, the prediction accuracy is calculated as

(11) $$\begin{array}{c} A=\frac{\mathrm{TP}+\mathrm{TN}}{N}. \end{array}$$

Precision score is defined as $P=\frac{\mathrm{TP}}{\mathrm{TP}+\mathrm{FP}}$, recall score is defined as $R=\frac{\mathrm{TP}}{\mathrm{TP}+\mathrm{FN}}$, and F1 score is defined as $F1=\frac{2*P*R}{P+R}$. As the F1 score is meant for binary classification, it needs some modification for multi-class classification. For multi-class classification, first, the class-wise F1 score is calculated and then the average is taken. The average F1 score is called the macro F1 score. From now on, wherever we mention the F1 score, we mean the macro F1 score. Matthew’s correlation coefficient is formulated as

(12) $$\begin{array}{c} \mathrm{MCC}=\frac{\mathrm{TP}\times \mathrm{TN}-\mathrm{FP}\times \mathrm{FN}}{\sqrt{\left(TP+FN)(TP+FP)(TN+FP)(TN+FN\right)}}. \end{array}$$

A value of +1 for MCC indicates the best agreement between the predicted and true values. In [51], the authors presented a case study where they demonstrated that the MCC value is best suited for classifiers with imbalanced data, i.e., the number of class labels is not the same for all classes.

## 3. Simulation Study Result

**Figure 4:**
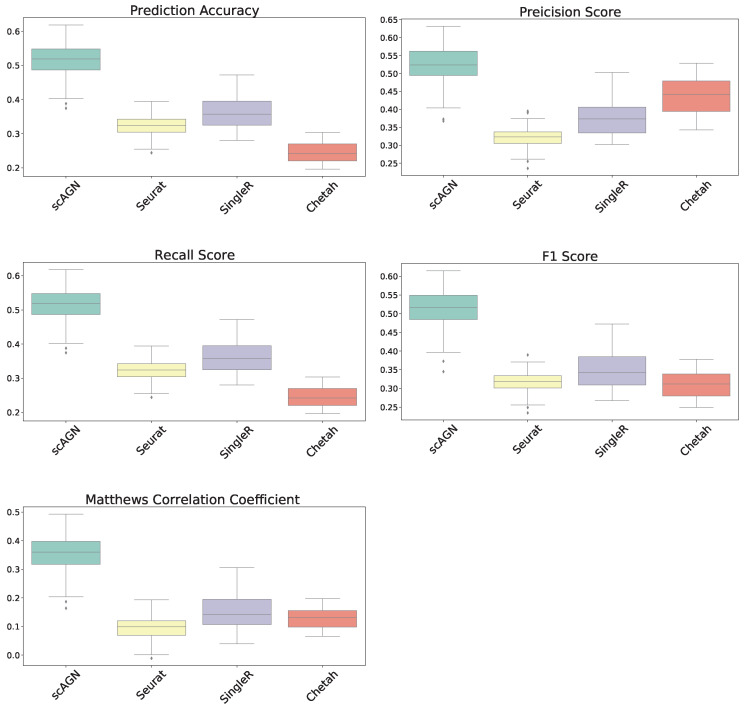
Boxplot to illustrate the performance of scAGN against other methods using simulated datasets with a sparsity of 95% (all four classes equiprobable). scMAP-cell and scMAP-cluster were not included in the plots as they failed for the datasets.

**Figure 5:**
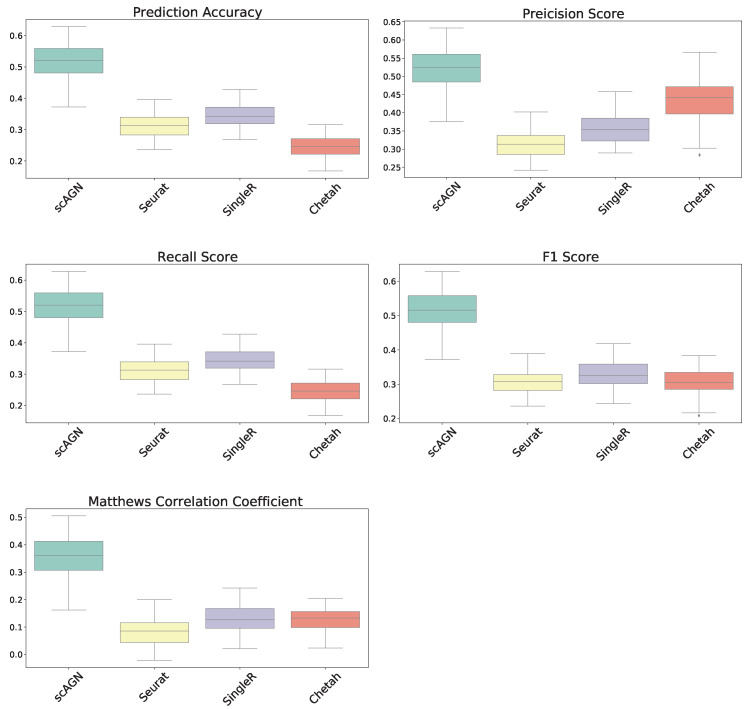
Boxplot to illustrate the performance of scAGN against other methods using simulated datasets with a sparsity of 95% when classes are imbalanced in the dataset. scAGN has superior performance compared to the existing methods.

In this section, our method is compared with five existing methods using the simulated datasets that are described in Section 2.1; namely, simulated datasets with 95% sparsity—four equiprobable classes (Figure 4), simulated datasets with 95% sparsity—four imbalanced classes (Figure 5), and 80% sparsity—four equiprobable classes (see the supplementary). The performance evaluation is based on five metrics; namely, prediction accuracy, precision score, recall score, F1 score, and Matthew’s correlation coefficient.

For datasets with a sparsity of 95% and four equiprobable classes, the boxplots show that the proposed method scAGN surpasses all the other methods in terms of all five evaluation metrics; Seurat came second while SingleR and Chetah were third and fourth, respectively; scMAP-cell and scMAP-cluster did not work with such high-sparsity datasets. For the simulated data with a sparsity of 80% and four equiprobable classes, the proposed method scAGN outperformed the existing methods (see Figure S1 in the Supplementary). We also conducted a simulation study where Splatter parameter de.prob was set to 0.5. Figure S2 in the Supplementary indicates that scAGN was superior to that of all baseline methods for this case as well.

Note that the scAGN selects the best neural network architecture out of a pre-determined set of hidden units and the number of layers. This requires several iterations. In our method, the number of hidden units were varied among 32, 64, 128, and 256, and the number of layers varied from 2 to 5. However, we find that our method scAGN is less stable compared to Seurat in general. This behavior is attributed to the random weight initialization of the neural network layer.

The performance of our method is also compared with other existing methods using simulated datasets with imbalanced classes. The boxplots of performance metrics, as shown in Figure 5, demonstrate that our method works best with simulated datasets on all performance metrics, even for the case of imbalanced classes.

## 4. Real Dataset Results

In this section, we first describe the performance of the scAGN method against baselines using all five metrics defined in Section 2.10 on real datasets. Next, we provide details of scAGN’s performance by looking at the confusion matrix of a few datasets as examples and the run-time complexity of our methods against baseline methods.

### 4.1. Label Propagation on Real Datasets

**Figure 6:**
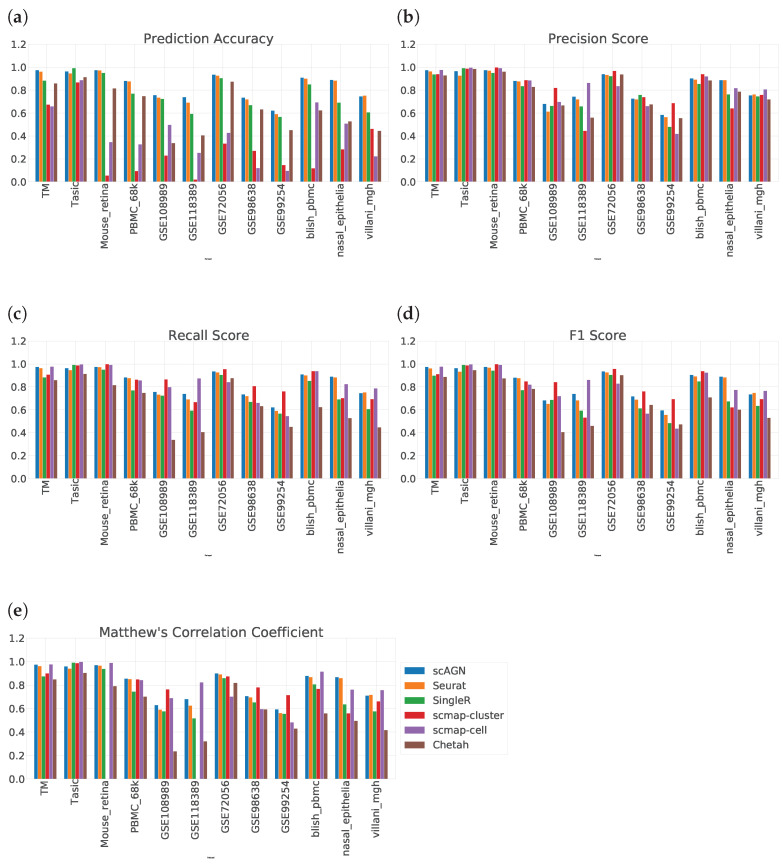
Performance evaluation of scAGN method against baseline methods using 12 real datasets. (**a**) We see that out of 12 datasets, scAGN performs best with 11 datasets. (**b**) In terms of precision score, there is no clear consensus on which method works best. However, the scAGN method performs 2nd best or 3rd best in all of the datasets. scMAP-cluster was best in 4 datasets out of 12 datasets. scMAP-cell was best in 5 datasets, while Seurat was best in only one dataset. Chetah and SingleR performed similarly. (**c**) Recall score for all six methods on 12 datasets. From the recall score also, we can see that there is no clear consensus on which method is the best. (**d**) F1 score also shows a similar trend as the recall score and our method is best in two datasets and second best or third best in the rest of the datasets. (**e**) From the MCC value, we see that scAGN is best in three datasets, while scmap-cell is best in four datasets.

We evaluate the ability of scAGN using real datasets. The performance of the classifier was evaluated on the prediction set as described in Section 2.5. On all real datasets, our method outperforms all baseline methods. Out of all baseline methods, Seurat V3 performs most similarly to our method. However, scMAP-cluster and scMAP-cell performed poorly with respect to our method. The results show that our method using a graph neural network performed consistently in all performance metrics: prediction accuracy, precision score, recall score, F1 score, and Matthew’s correlation coefficient (MCC) (Figure 6).

The result of prediction accuracy is summarized in Figure 6a. In 11 out of 12 datasets, our method performed best in terms of prediction accuracy. For a dataset with imbalanced classes, i.e., not all classes have the same number of samples, prediction accuracy is not enough. In that case, we also need to assess the performance of the classification method on other metrics such as precision score, recall score, F1 score, and MCC. The precision score tells us how many positive predictions were made. Recall tells us how many of the positive cases the classification method predicted correctly, and overall the positive cases in the dataset. The F1 score is the harmonic mean of the precision score and recall score. MCC provides a reliable statistical measure in the case of datasets with imbalanced classes as it considers true positives, false negatives, true negatives, and false positives, proportionally both to the size of positive elements and the size of negative elements in the dataset.

The precision score, recall score, F1 score, and MCC comparing all datasets are given in Figure 6b–e. From Figure 6b, we see that there is no clear consensus on which method works best. However, scmap-cluster was best in 4 out of 12 datasets; our method was 2nd best or 3rd best in all 12 datasets. A similar trend was observed in the recall score bar plot in Figure 6c as well as in the F1 score barplot in Figure 6d. From Figure 6e, we see that scAGN’s performance was best in three datasets, second best in five datasets, and third best in the remaining datasets.

One thing to note here is that scMAP-cluster and scMAP-cell also predict “unassigned” for samples that cannot be predicted accurately. However, to calculate the precision, recall, and F1 scores, the number of classes is required to be the same in true label sets and predicted label sets. As a remedy, we remove all samples for which scMAP-cell and scMAP-cluster predicted “unassigned” to calculate the precision, recall, and F1 scores, and MCC.

### 4.2. Confusion Matrix

**Figure 7:**
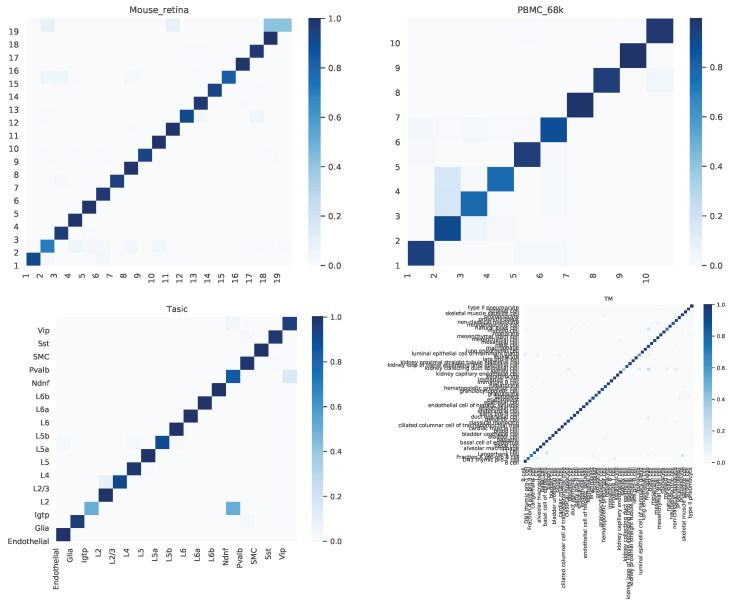
Confusion matrix for healthy cells as examples. We observe that the proportions of cells incorrectly predicted are very low and the overall predictive power of scAGN is very high. The only notable incorrect prediction is the Tasic dataset, where roughly 50% of Pvalb cell types were incorrectly classified as L2 cell types.

We further dived deep into the performance of scAGN for multiple datasets. A confusion matrix provides a summary of prediction results by a classifier in visual form as shown in Figure 7. The confusion matrix summarizes the number of correct and incorrect predictions, which can be analyzed to understand the predictive power of the classifier. We look at the confusion matrix of healthy cells. We find out that the proportions of cells incorrectly predicted are very low, and the overall predictive power of scAGN is very high. The only notable incorrect prediction is the Tasic dataset, where roughly 50% of Pvalb cell types were incorrectly classified as L2 cell types.

### 4.3. Impact of Hidden Units and Number of Layers

**Figure 8:**
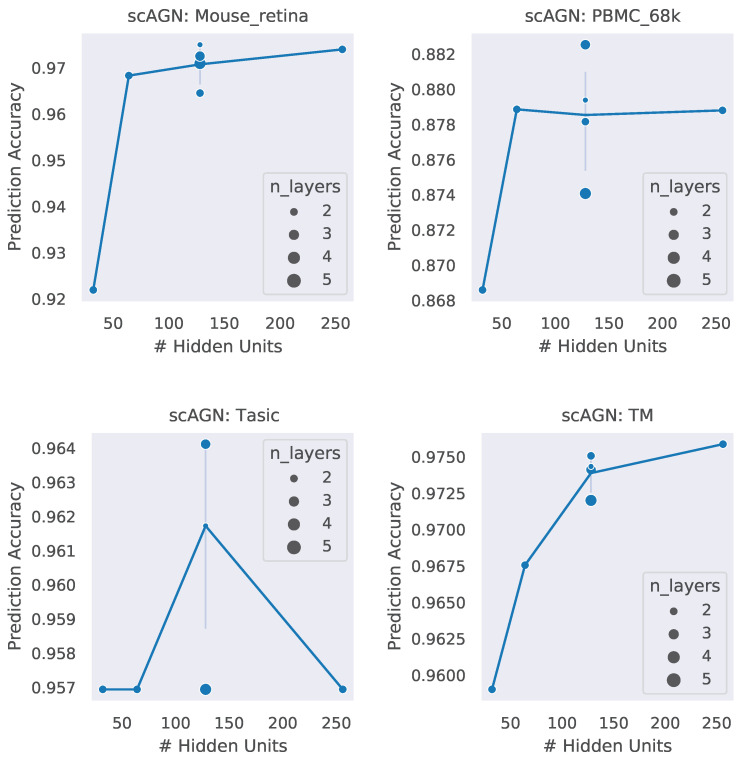
Prediction accuracy with the number of hidden units and the number of layers for healthy cells. The number of hidden units used were 32, 64, 128, and 256. The number of layers were varied from 2 to 5. The size of the bubble represents the number of layers used in the graph neural network. For a fixed hidden unit, the varying number of layers does not provide a definite conclusion on a trend of prediction accuracy with the number of layers. Lines were drawn passing through the average of the number of layers where the number of hidden units is fixed. With the Tasic dataset, we see an exception where the trend of prediction accuracy with the number of hidden units is inconclusive.

**Figure 9:**
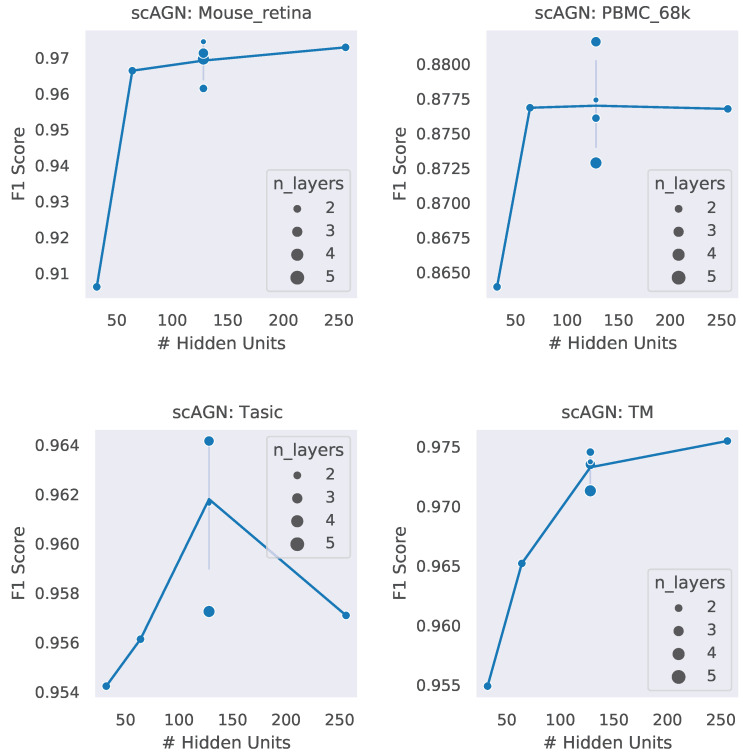
F1 score with the number of hidden units and the number of layers. The number of hidden units used were 32, 64, 128, and 256. The number of layers were varied from 2 to 5. The size of the bubble represents the number of layers used in the graph neural network. For fixed hidden units, varying the number of layers does not provide a definite conclusion on a trend of the F1 score with the number of layers. Lines were drawn passing through the average of the number of layers where the number of hidden units is fixed. With the Tasic dataset, we see an exception where the trend of the F1 score with the number of hidden units is inconclusive.

A neural network architecture plays a crucial role in determining the predictive power of a neural network classifier. Finding the best neural architecture is important for the superior performance of a neural network classifier. For scAGN, a neural network classifier, hidden units, and the number of layers matter. While we did not perform any hyperparameter search for the best neural network, we varied the number of hidden units and the number of layers to study the trend of performance with hyperparameters such as the number of hidden units and the number of layers. We visualize the impact of the number of hidden units and the number of layers on the prediction accuracy and F1 score for healthy cells (Figure 8 and Figure 9). The impact of the number of hidden units and the number of layers on these two performance metrics for all 12 datasets can be seen in Figures S3 and S4 in the Supplementary. In general, we found that, with an increase in the number of hidden units, the prediction accuracy as well as F1 score improve as we increase the number of neurons. Having a too little number of hidden units can cause underfitting, while it should also be noted that using too many hidden units can cause overfitting and the model becomes less generalizable. Increasing the number of layers did not have a significant impact on the performance metrics of the scAGN classifier.

### 4.4. Runtime Complexity

**Figure 10:**
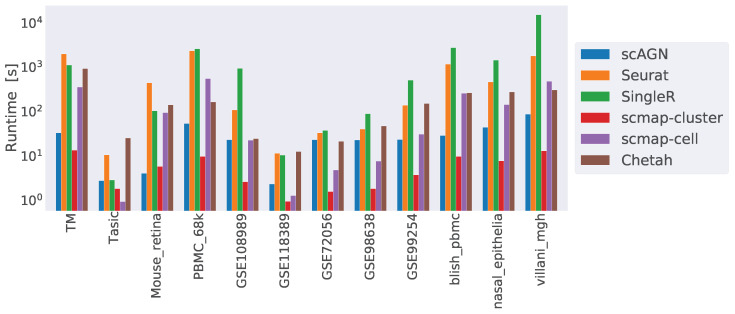
Runtime complexity of various classifiers included in our study. While scMAP-cluster was the fastest method, scAGN came second. SingleR was the slowest method. The y-axis is log-scaled for clarity.

Finally, we compared the runtime complexity of our method against other scRNAseq classifiers. We ran all our methods on a 64-bit Ubuntu 18.04 machine with 256 GB of RAM and processor Intel Core i9-10900X CPU with a CPU cycle of 3.70 GH and 20 cores. In addition to that, the machine had an NVIDIA GeForce RTX 3090 graphics card with a graphics memory of 4096 MB. For simulated datasets containing 1000 cells and 800 genes, scAGN on average took 20 s to finish, while Seurat took 7 s, SingleR and Chetah each took 5 s, and scMAP-cell and scMAP-cluster each took roughly 1 s. From the analysis of the runtime complexity, we find that runtime increased with the number of cells and genes present in the dataset. For example, COVID-19 datasets were the largest ones and took the maximum time to finish the training and prediction. scAGN’s runtime complexity was the second lowest, with scMAP-cluster being the fastest method. Please note that the run-time estimate of scAGN is slightly higher due to the minimum 2000 steps that are used as early stopping criteria. In such a case, even if no improvement to training loss is made, training continues until 2000 steps have passed. A summary of the run time complexity of all methods is provided in Figure 10.

## 5. Discussion and Conclusions

scAGN is a flexible approach that can potentially improve the accuracy and robustness in identifying cell types at the single cell level. In terms of clinical practices, the ability to accurately identify cell types in scRNAseq data can help the understanding of various diseases and the development of targeted therapies. For example, in cancer research, the identification of different cell types within a tumor can provide insights into the underlying mechanisms of the disease and help to identify potential therapeutic targets. Similarly, in neuroscience, the identification of specific cell types in the brain can provide insights into the underlying mechanisms of neurological disorders and help to develop more effective therapies.

From our simulation study, we find that our method works best with highly sparse data compared to other methods. Other methods such as Seurat create models based on anchor points, while scMAP-cell and scMAP-cluster require projecting cells onto a known cluster or another labeled cell. SingleR finds the top correlated cell types. Such methods fail when a dataset is highly sparse. Model-fitting-based algorithms need to fit more coefficients, while tree-building methods such as Chetah need to account for greater depth to consider all features. In scAGN, we deal with these issues at the feature processing level discussed in Section 2.5, which makes scAGN suitable for training on sparser datasets.

We notice that, although real datasets have the same level of sparsity as a simulated dataset, the performance of our method is almost the same as other methods in the case of real datasets. However, with the simulated datasets, our method outperforms other methods. The reason behind this outcome may be due to the fact that, in simulations, there is a stronger statistical relationship between various cells samples simulated while such a stronger relationship among various cell samples in real datasets may not exist due to variability in sequencing steps, lab experiments, and other external factors. We need further investigation on improving the performance of graph-based methods for real datasets. One key approach might be a hyperparameter search of neural network parameters exhaustively for the best neural network architecture. However, such steps need greater computing requirements.

In this study, we propose a novel method of cell-type detection based on transductive learning using an attention-based graph neural network. The scAGN exploits higher-order topology to create a neural network classifier. We compared the performance of our method with five other methods and, in terms of prediction accuracy, our method was superior while, in terms of precision, recall, and F1 scores, no consensus was reached on the superiority of any of the methods mentioned in this study.

One key aspect to note is that our work establishes a topological relationship based on information present in the data. However, a better topological relationship may be captured based on external data such as gene–gene interaction networks, splicing information, and metadata about cell samples. In our future work, we hope to include external information in creating a graph neural network for cell-type classification of single-cell data. Further, a natural extension would be to assess our methods on other single-cell modalities such as scATAC-seq and CITE-seq.

## Figures and Tables

**Table 1:** Real datasets used for comparing scAGN’s performance with baseline methods.

Name	#Cells	#Genes	#Classes	Protocol	Species	Tissue	Sparsity
Mouse_retina	27,499	2000	19	Drop-seq	Mus musculus	Retina	93.43%
PBMC_68k	68,579	2000	10	10x	Homo sapiens	PBMC	97.25%
Tasic	1679	2000	17	SMARTer	Mus musculus	Visual Cortex	71.78%
TM	54,865	2000	55	10x	Mus musculus	Multiple	93.98%
GSE108989	11,138	23,459	5	SMART-Seq2	Homo sapiens	Colorectal Cancer	84.86%
GSE118389	1534	21,785	6	SMART-Seq2	Homo sapiens	Breast Cancer	89.63%
GSE72056	4513	23,690	7	SMART-Seq2	Homo sapiens	Melanoma Tumors	81.38%
GSE98638	5063	23,459	12	SMART-Seq2	Homo sapiens	Liver Cancer	85.17%
GSE99254	12,346	23,459	19	SMART-Seq2	Homo sapiens	Lung Cancer	87.49%
blish_pbmc	44,721	26,361	16	Seq-Well	Homo sapiens	PBMC	96.25%
nasal_epithelia	32,588	32,871	17	Seq-Well	Homo sapiens	Nasal Epithelia	95.13%
villani_mgh	59,506	24,179	16	10xV2	Homo sapiens	PBMC	94.37%

## Data Availability

The dataset used in this study has been made available publicly through Cyverse at https://doi.org/10.25739/fgh0-2d05 (accessed on 25 December 2022). The computer code to implement the scAGN method can be downloaded from https://github.com/anlingUA/scAGN (accessed on 25 December 2022).

## Supplementary Materials

The supporting information can be downloaded at: https://www.mdpi.com/article/10.3390/genes14020506/s1, Figure S1: Boxplot to illustrate the performance of scAGN against other baseline methods using simulated datasets with sparsity of 80%; Figure S2: Boxplot to illustrate the performance of scAGN against other baseline methods using simulated datasets with sparsity of 95–97%; Figure S3: Prediction accuracy with the number of hidden units and number of layers for all 12 real datasets; Figure S4: F1 score with the number of hidden units and number of layers for all 12 real datasets.
