# Supplementary material for: Attention-Based Graph Neural Network for Label Propagation in Single-Cell Omics
Source: Genes (Basel). 2023 Feb 16;14(2):506. doi: 10.3390/genes14020506 (PMC9957137; doi:10.3390/genes14020506)
Supplement: Supplementary file 1 [file genes-14-00506-s001.zip › genes-2156521-supplementary.pdf]

Supplementary Materials: Attention-Based Graph Neural Network for Label Propagation in Single-Cell Omics

Rahul Bhadani, Zhuo Chen, Lingling An

In this supplementary material, we describe the performance of our method scAGN on additional simulated datasets. In addition to that, we also look at the impacts of hidden units and the number of layers on performance metrics.

1 Results and Discussion on Simulation Study

To generate simulated cell-gene count matrices for the scRNAseq dataset, we use the Splatter package. The splatter package provides several parameters that can be tuned to create simulated scRNAseq data with desired properties such as the number of classes (or cell types), class imbalance, sparsity level, the number of cells, genes, expression levels, technical noise, etc. This can be useful for testing and evaluating scRNA-seq analysis methods, or for training machine learning models on simulated data. For completeness, we provide a description of a few parameters we used for the generation of the simulated dataset in the simulation study:

- `de.prob`: It specifies a probability that a gene can be differentially expressed in a group.
- `dropout.mid`: It specifies the midpoint parameter for the dropout logistic function that is used to induce dropout in the synthetic dataset.
- `dropout.shape`: It specifies the shape parameter for the dropout logistic function that is used to induce dropout in the synthetic dataset.

1.1 Sparsity of 95%

In the main text, we discussed results using a simulated dataset with a sparsity of 95%. We generated 50 replicates of simulated datasets with a sparsity of 95%. Each simulated dataset contains 1,000 cells and 800 genes with equiprobable 4 cell types. `dropout.mid` value was chosen to be 7 for all 50 replicates. `dropout.shape` was -1 for all fifty replicates which is the default value in the Splatter package. Further, an additional parameter `de.prob` was set

to 0.2 which is the probability of genes being differentially expressed. Similar settings were used for simulated datasets with imbalanced classes.

1.2 Sparsity of 80%

In this section we evaluate the performance of scAGN on 50 replicates of simulated datasets with 80% sparsity where `de.prob` set to 0.2. Each simulated dataset contains 1,000 cells and 800 genes with equiprobable 4 cell types.

The performance of all methods on simulated datasets with 80% sparsity is illustrated in Figure S1 via boxplot. Only the method Chetah is comparable with scAGN for the precision metrics. In all other performance metrics, the new method scAGN is superior among all the methods.

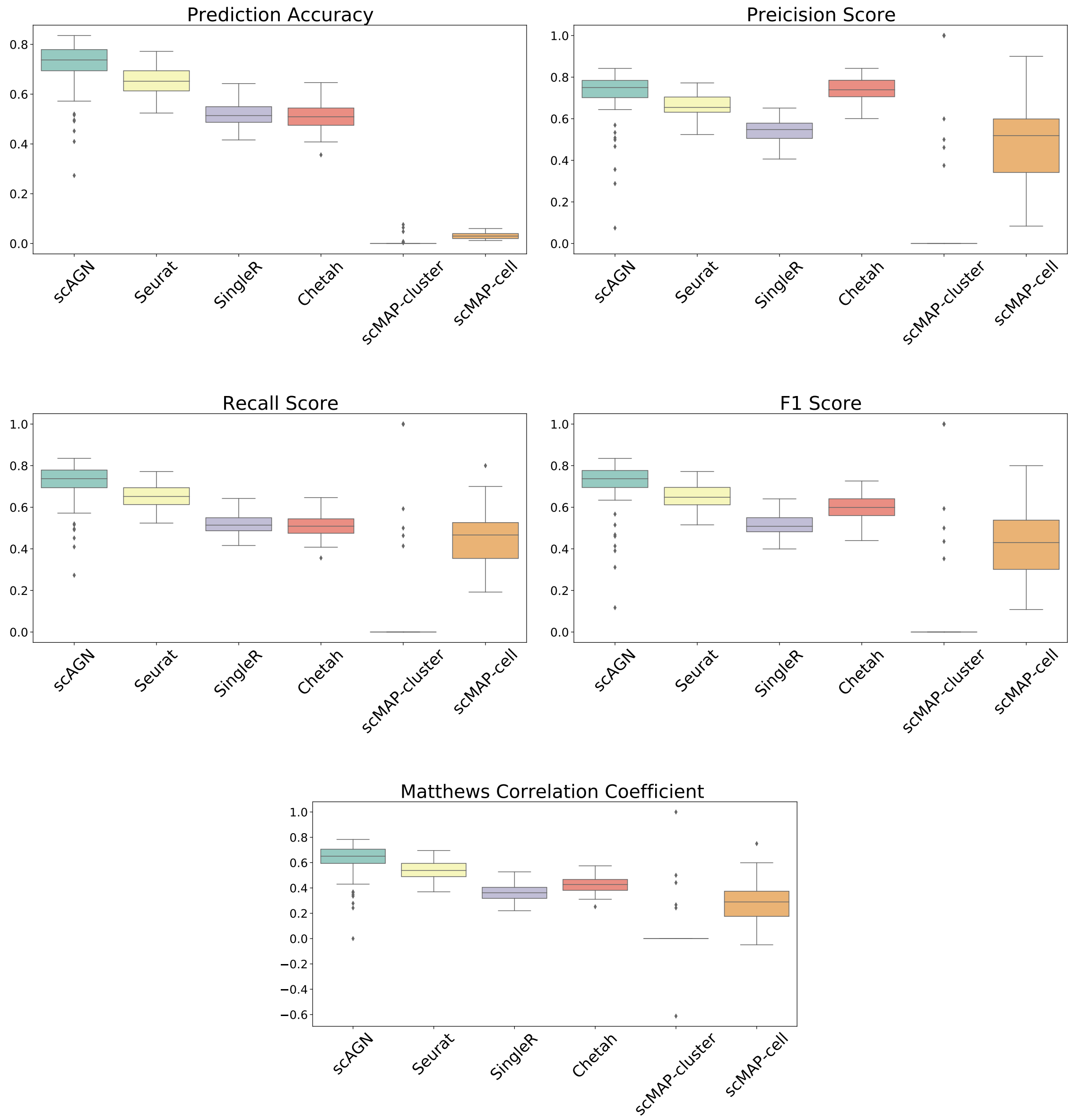

Figure S1: Boxplot to illustrate the performance of scAGN against other baseline methods using simulated datasets with sparsity of 80%. We note that scAGN's performance is better overal although Seurat's performance was relatively stable compared to our method. SingleR and Chetah performed similar while scMAP-cell and scMAP-cluster failed to identify cell types for most cell samples.

1.3 `de.prob` set to 0.5

This section provides a comparison of our method against baseline methods using simulated datasets where datasets were generated using Splatter by `de.prob` set to 0.5 which is the probability of genes being differentially expressed. We use simulated datasets with the sparsity 95% which were generated using `dropout.mid` values of 4 and 7 respectively. Overall performance of scAGN was superior to that of all baseline methods for datasets with a sparsity of 95%. Boxplot illustrating performances of all methods are provided in Figure S2.

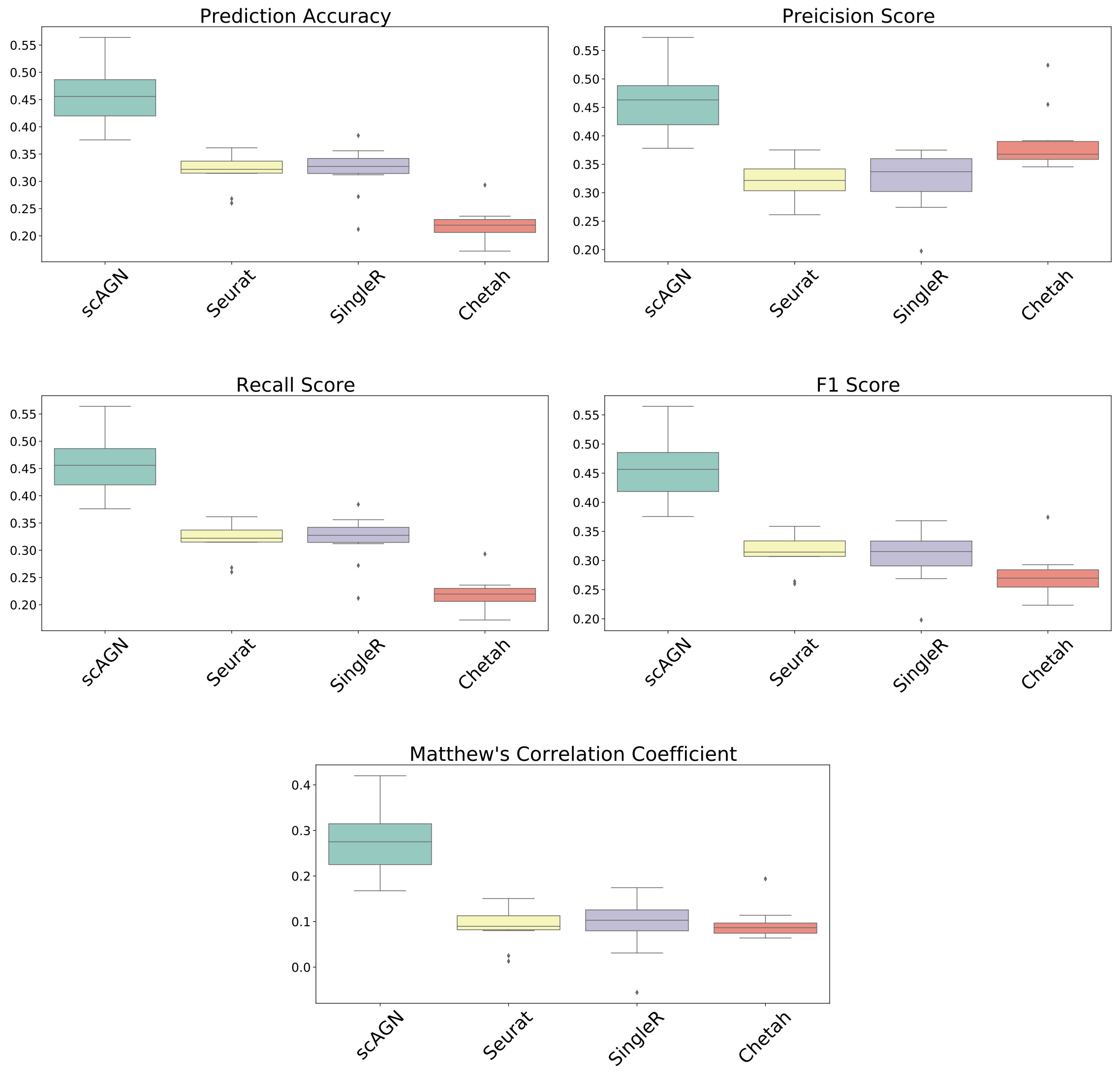

Figure S2: Boxplot to illustrate the performance of scAGN against other baseline methods using simulated datasets with sparsity of 95%-97%. The simulated dataset was generated by setting `de.prob`=0.5 in Splatter. We see that for datasets with such high sparsity, scAGN's performance was superior and consistent for all four metrics. Seurat came second while SingleR and Chetah were third and fourth respectively. However, scMAP-cell and scMAP-cluster didn't work with datasets having such high sparsity. Hence, we didn't provide boxplots for these two methods.

2 Impact of Hidden Units and Number of Layers

A neural network architecture plays a crucial role in determining the predictive power of a neural network classifier. Finding the best neural architecture is important for the superior performance of a neural network classifier. For scAGN, a neural network classifier, hidden units, and the number of layers matter. While we didn't perform any hyperparameter search for the best neural network, we varied the number of hidden units and number of layers to study the trend of performance with hyperparameters such as the number of hidden units and the number of layers. We visualize the impact of the number of hidden units and the number of layers on performance metrics for a few datasets in Figure S3 and Figure S4. We only provide the impact of hidden units and the number of layers for prediction accuracy and the F1 score only for brevity as the F1 score captures the trend seen in the recall score and precision score. A similar trend was observed in recall score, precision score, and MCC. In general, we found that with an increase in the number of hidden units, prediction accuracy, as well as F1 score, improves. Increasing the number of layers didn't have a significant impact on the performance metrics of the scAGN classifier.

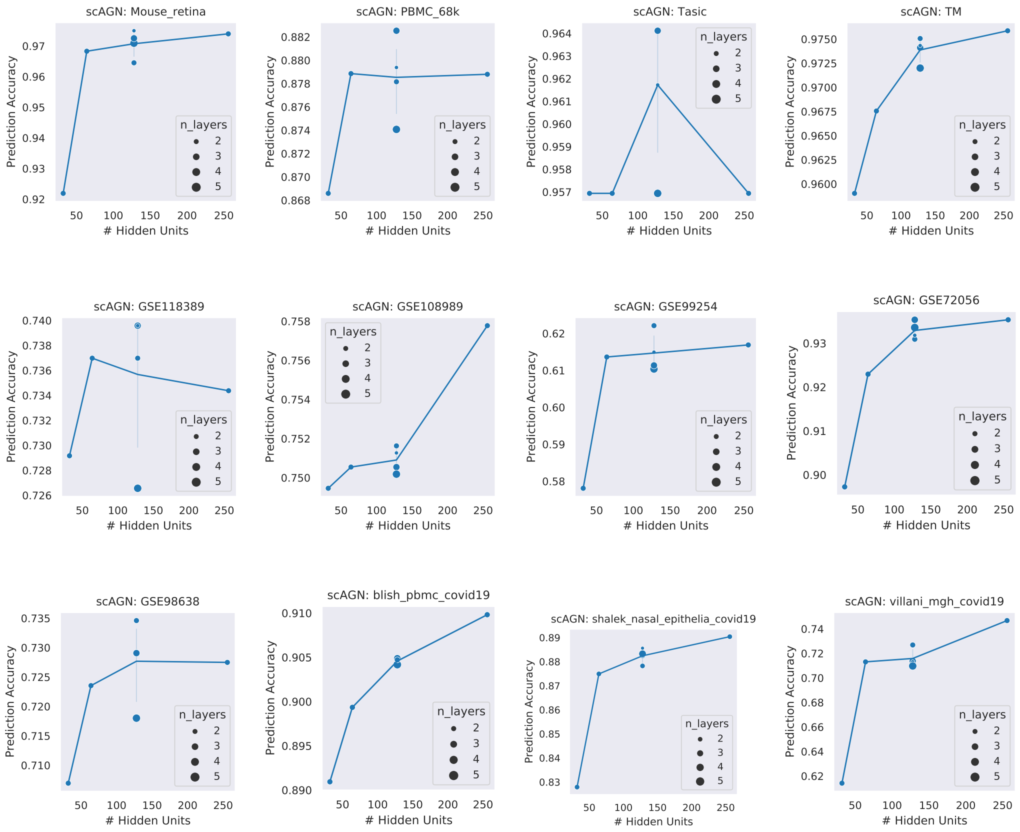

Figure S3: Prediction accuracy with the number of hidden units and number of layers for all 12 real datasets. The number of hidden units used were 32, 64, 128, and 256. The number of layers were varied from 2 to 5. Size of the bubble represents the number of layers used in the graph neural network. For a fixed hidden units, varying number of layers doesn't provide a definite conclusion on a trend of prediction accuracy with the number of layers. Lines were drawn passing through the average of number of layers where the number of hidden units is fixed. With Tasic dataset, we see an exception where trend of prediction accuracy with the number of hidden units is inconclusive. Similarly, with GSE118389 dataset, we see an exception where trend of prediction accuracy with the number of hidden units is inconclusive.

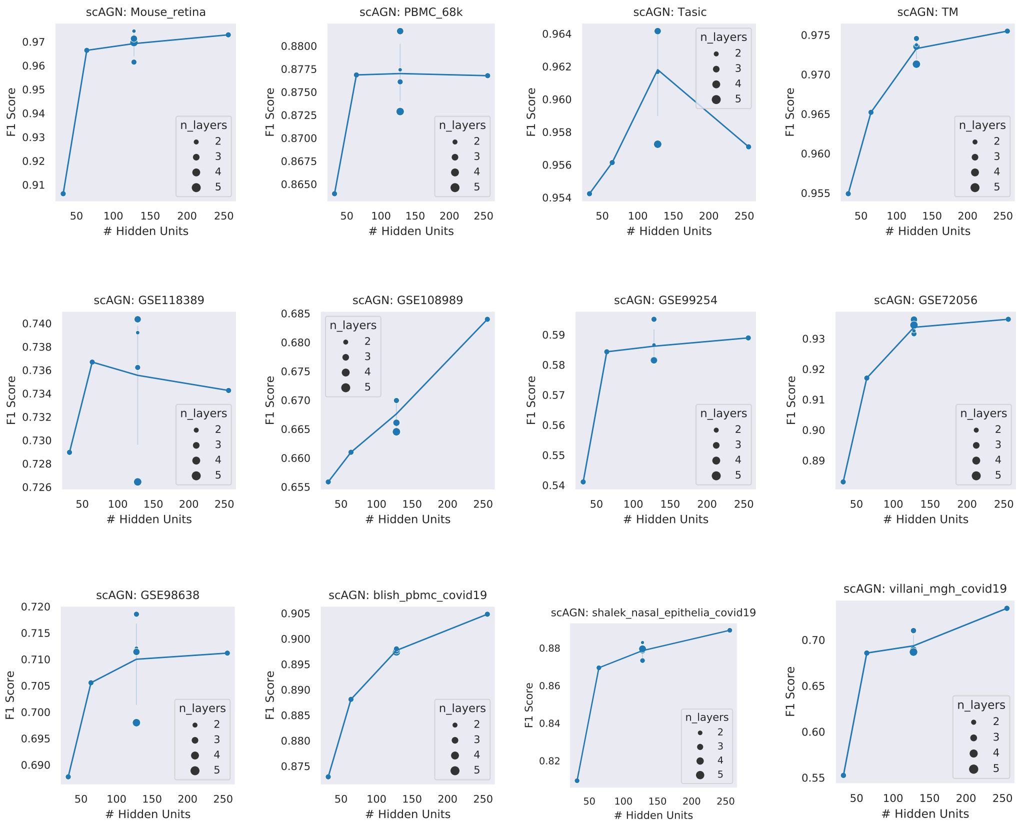

Figure S4: F1 score with the number of hidden units and number of layers for all 12 real datasets. The number of hidden units used were 32, 64, 128, and 256. The number of layers were varied from 2 to 5. Size of the bubble represents the number of layers used in the graph neural network. For a fixed hidden units, varying number of layers doesn't provide a definite conclusion on a trend of F1 score with the number of layers. Lines were drawn passing through the average of number of layers where the number of hidden units is fixed. With Tasic dataset, we see an exception where trend of F1 score with the number of hidden units is inconclusive. Similarly, with GSE118389 dataset, we see an exception where trend of F1 score with the number of hidden units is inconclusive.
